# Cognitive biases as an adaptive strategy in autism and schizophrenia spectrum: the compensation perspective on neurodiversity

**DOI:** 10.3389/fpsyt.2023.1291854

**Published:** 2023-12-04

**Authors:** Marcin Rządeczka, Maciej Wodziński, Marcin Moskalewicz

**Affiliations:** ^1^Institute of Philosophy, Maria Curie-Sklodowska University in Lublin, Lublin, Poland; ^2^IDEAS NCBR, Warsaw, Poland; ^3^Philosophy of Mental Health Unit, Department of Social Sciences and the Humanities, Poznan University of Medical Sciences, Poznań, Poland; ^4^Phenomenological Psychopathology and Psychotherapy, Psychiatric Clinic, University of Heidelberg, Heidelberg, Germany

**Keywords:** neurodiversity, diametrical model of autism and psychosis, evolutionary psychiatry, computational psychiatry, cognitive biases, autism spectrum, schizophrenia spectrum, temporal experience

## Abstract

This article presents a novel theoretical perspective on the role of cognitive biases within the autism and schizophrenia spectrum by integrating the evolutionary and computational approaches. Against the background of neurodiversity, cognitive biases are presented as primary adaptive strategies, while the compensation of their shortcomings is a potential cognitive advantage. The article delineates how certain subtypes of autism represent a unique cognitive strategy to manage cognitive biases at the expense of rapid and frugal heuristics. In contrast, certain subtypes of schizophrenia emerge as distinctive cognitive strategies devised to navigate social interactions, albeit with a propensity for overdetecting intentional behaviors. In conclusion, the paper emphasizes that while extreme manifestations might appear non-functional, they are merely endpoints of a broader, primarily functional spectrum of cognitive strategies. The central argument hinges on the premise that cognitive biases in both autism and schizophrenia spectrums serve as compensatory mechanisms tailored for specific ecological niches.

## Introduction: the concept of neurodiversity as the intersection of computational and evolutionary approaches to autism and schizophrenia spectrum

The concept of neurodiversity recognizes and respects neurological differences as any other human variation. These variations include Dyslexia, Attention Deficit Hyperactivity (ADH), Autism Spectrum (AS), and others. From the standpoint of evolutionary biology, neurodiversity can be seen as a result of the complex interplay between genetic variation, environmental adaptation, and natural selection. The existence of diverse neurological profiles within human populations might reflect a form of balancing selection, where different cognitive styles and abilities are maintained in the population because they offer various adaptive advantages (1).

The concept of neurodiversity, which respects neurological differences as natural variations among humans, is well-suited to be explored through a framework that integrates the evolutionary principles of natural selection and the analytical methods of modern computational psychiatry. While the evolutionary angle traces the historical and genetic underpinnings, the computational model unravels the neural and cognitive mechanisms. The evolutionary framework posits that autism and schizophrenia exist on opposite ends of a cognitive and social spectrum. While autism is characterized by a hyper-systemizing cognitive style, schizophrenia leans toward hyper-mentalizing. Hyper-systematizing is the heightened ability or tendency to identify and analyze patterns, systems, and rules in the external world. This cognitive style explains preference for consistent and rule-based information (2, 3). Hyper-mentalizing refers to the excessive attribution of mental states, intentions, or beliefs to others, often leading to incorrect interpretations of social situations. This cognitive bias explains why individuals might infer elaborate intentions or beliefs in others that are not grounded in reality (4, 5). The combination of both perspectives reinforces this model, drawing parallels and contrasts between the two conditions.

Autism is often misunderstood and surrounded by numerous stereotypes that oversimplify or misrepresent the condition (6). Contrary to some negative stereotypes, specific neurological differences associated with AS may have provided adaptive advantages in specific environmental contexts (7). For example, a strong focus on systematizing might have been beneficial in early human societies for tasks requiring attention to detail, such as toolmaking or tracking prey (8). The terms “Autism Spectrum Disorder” (ASD) and “Autism Spectrum Condition” (ASC) have faced criticism and a shift in perception over recent years. The word “disorder” in ASD can be viewed as pathologizing, suggesting something inherently wrong or dysfunctional, which may further stigmatize individuals within the spectrum. In contrast, the increasing acceptance of neurodiversity emphasizes the idea that autistic individuals have a different but equally valid neurological configuration, and as such, many advocates and experts are pushing for language that does not inherently label these differences as problematic or inferior. The term Autism Spectrum (AS) is free of misleading connotations (9). It does not connote a “problem” and thus helps to promote acceptance, understanding, and respect for those on the autism spectrum.

Similarly, subclinical schizotypy (schizotypal traits not severe enough to warrant a clinical diagnosis) can manifest in ways that might have adaptive advantages. Individuals displaying certain schizotypal traits might have played significant roles in religious and shamanic rituals. Magical thinking, a common trait in schizotypy, refers to connecting unrelated events, leading to the belief in unseen forces or causality. These traits can promote a shared belief system within a community and reinforce group norms and values, which may foster unity and trust (10).

The genetic diversity underlying neurodiversity may also reflect the influence of sexual selection, where certain cognitive or behavioral traits become desirable mating characteristics (11). Environmental factors and epigenetics may shape neurodiversity, with different neurological profiles being adaptive in various ecological niches (12). The concept of neurodiversity aligns with cognitive niche construction by emphasizing that neurological differences are natural variations in human cognition and behavior rather than pathologies (13). An ecological niche in this context is a set of environmental conditions, challenges, and opportunities within which a particular cognitive profile confers specific adaptive advantages or disadvantages. This encompasses both the physical environment (such as geographic and climatic features) and the social environment (including cultural, economic, and community structures) that interact with the cognitive and perceptual capabilities of individuals. For those on the autism and schizophrenia spectra, an ecological niche is where their unique cognitive processing style (detail-focused or pattern-seeking in autism or highly inferential and context-sensitive in schizophrenia) best align with the tasks, roles, and demands of that environment, allowing individuals to thrive or fulfill essential societal functions.

Neurodiversity thus celebrates differences and recognizes that they offer unique ways of perceiving and interacting with the world. On the other hand, society’s implicit stereotypes—viewing neurological differences as deficits—can create maladaptive developmental niches (6). In essence, neurodiversity advocates for reshaping these niches by challenging these stereotypes. It fosters environments that acknowledge and leverage the distinct cognitive strengths of neurodivergent individuals, thereby leading to a more inclusive and adaptive societal framework (14). By harnessing datasets from cognitome to phenome, the Nomothetic Network Psychiatry (NNP) approach, akin to evolutionary computational psychiatry, aspires to revolutionize psychiatric diagnosis and treatment, bridging existing fragmentation with a unified, data-driven lens that challenges conventional diagnostic criteria and potentially resolving its ongoing crisis (15–17).

## Evolutionary perspective on mental disorders

An evolutionary perspective is indispensable for understanding psychiatric conditions that often originate from complex and multifaceted antecedents. It can illuminate human vulnerability to mental disorders and offer insights into the persistence of certain maladaptive traits or behaviors within the genetic pool (18). Recognizing the role of evolutionary history in contemporary susceptibility to psychiatric conditions can potentially pave the way for more potent and effective computational models. For example, historically viewed primarily as a maladaptive pathology, mild to moderate anxiety can be understood as a protective mechanism that evolved to help our ancestors detect and avoid potential threats. In the ancestral environment, a heightened sense of alertness and caution might have safeguarded individuals from predators or other dangers, thus increasing their chances of survival and reproduction (19). Similarly, mild depression coupled with social withdrawal might have minimized conflicts within tribal groups, and reduced energy levels could have conserved resources during times of scarcity or promoted reflection during crises (20).

Additionally, the scope of evolutionary theory extends beyond disease vulnerabilities to encapsulate human uniqueness, focusing on the role of cumulative culture and gene-culture co-evolution in shaping human physiology and cognition (21). The interplay between genetic evolution and cultural shifts has extensively influenced our physical and cognitive attributes, forming the cornerstone of human uniqueness.

Disease and disorder persistence can be traced across multiple evolutionary pathways. These include mismatch phenomena, life history theory, overactive defenses, evolutionary arms races between pathogens and hosts, constraints imposed by evolutionary history, sexual selection outcomes, balancing selection and heterozygote advantage, demographic history, natural selection prioritizing reproductive success, and the presence of deleterious alleles (22).

Evolutionary psychiatry does not aspire to replace mainstream psychiatry; instead, it seeks to augment traditional perspectives by infusing new insights into the genesis, progression, and treatment of mental disorders (23). The principal goal is to integrate evolutionary understanding into computational models, fostering a holistic understanding of psychiatry. Computational methods allow for the modeling and simulation of complex genetic, neural, and behavioral systems, enabling researchers to explore how various neurological profiles might have evolved (24). These models can incorporate data from genetics, neuroscience, and psychology to provide a holistic view of the factors influencing neurodiversity (25). Recognizing the evolutionary roots of neurological differences can tailor treatment plans to the individual’s unique cognitive and behavioral profile (26). Moreover, factors such as the extremes of functional adaptations, cultural evolution, and gene-culture coevolution contribute significantly to this comprehensive conception (27). Each of these elements is integral in understanding why certain conditions persist despite their apparent disadvantageous nature. Integrating computational psychiatry with evolutionary psychology creates a potent capable of deciphering neurodiversity. Exploring evolutionary origins and consequences of these conditions can cultivate a more holistic, integrative, and effective approach to contemporary psychiatry (28).

## Understanding the diametrical model of autism and schizophrenia

The diametrical model (DM) of autism and schizophrenia presents these two psychiatric conditions as representing opposite ends of a cognitive and behavioral spectrum. It has emerged from observing contrasting phenotypic traits and underlying genetic factors between them.

Autism is a neurodevelopmental condition classified in diagnostic manuals as developmental disorder and characterized by difficulties/atypicalities in social interaction and (verbal and non-verbal) communication, as well as restricted and repetitive patterns in behaviors, interests, and activities. In contrast, schizophrenia is often marked by disorganized thinking, hallucinations, delusions, and an exaggerated preoccupation with social interaction. DM proposes that these contrasting phenotypes reflect underlying antagonistic processes. While individuals with autism often struggle with the ability to understand mental states of some neurotypical people, those with schizophrenia may exhibit hyper-mentalizing, where the capacity for empathy is exaggerated to the point of generating delusional thoughts about others’ intentions (8, 29). Moreover, autistic individuals typically show atypicalities in language development and some may struggle with metaphorical or abstract language. In contrast, schizophrenic individuals may exhibit disorganized speech and engage in metaphorical thinking to an extreme degree (30). While DM centers on the mentalistic–mechanistic trade-off, it also provides a broader context for by-product models that link autism risk to specific cognitive profiles (e.g., enhanced visuospatial skills) and developmental patterns (e.g., delayed behavioral and neurological development; 31). The term “trade-off” generally refers to an exchange where an increase in one trait is coupled with a decrease in another, reflecting a balance of benefits and costs. The mentalistic-mechanistic trade-off implies that an evolutionary advantage in one cognitive domain might come at the expense of another. This concept in DM is distinct from trade-offs discussed in the context of model building or algorithm design, where trade-offs might involve the complexity of a model vs. its interpretability or the precision vs. speed in computational tasks. However, it shares the underlying principle that optimizing one aspect involves compromise in another.

Research has also identified genetic factors that may underlie this diametrical relationship. Some genetic variations associated with an increased risk for autism are related to a decreased risk for schizophrenia, and vice versa (32). For example, certain copy number variants (CNVs), which involve duplications or deletions of DNA segments, have been found to have opposing effects on the risk of autism and schizophrenia (33). There are also differential parental age effects on the risks for both spectra, with advanced paternal age being associated with an increased risk of autism and advanced maternal age being linked to an increased risk of schizophrenia (30).

DM also posits that autism and schizophrenia represent opposite extremes of cognitive and social behavior, paralleling the r-selected (fast, reproductive-focused) vs. K-selected (slow, survival-focused) evolutionary strategies of the life history theory. Autism aligns more with K-selected strategies emphasizing systemized thinking and routine, while schizophrenia aligns with r-selected strategies emphasizing social cognition and fluidity (34). The correlates of autistic-like traits include muted sex drive and restricted sociosexuality, preserved or heightened interest in romantic relationships, and increased investment of time and resources in long-term partners—all traits that promote parental investment at the expense of mating effort (35).

## Autism and systematizing: some subtypes of autism are a unique cognitive strategy to manage cognitive biases at a price of fast and frugal heuristics

Autism is recognized as a heritable neurodevelopmental state notorious for its deleterious impact on reproductive success. This raises an intriguing question regarding its persistence in the gene pool despite natural selection (36). Genes associated with autism are not recently evolved; they demonstrate antique characteristics, including large gene and protein sizes and regulatory sequences facilitating control over gene expression (37). A striking 1% prevalence of AS in the UK (38) suggests potential adaptive properties of autism, despite its reproductive disadvantages. Some evidence suggests that at least 3–10% of autism cases relate to *de novo* and rare genetic variants (39). From the evolutionary standpoint, however, some forms of autism (in older literature often described as “hyper-functioning”) can be seen as adaptive. It is highly heritable and stems from multiple genes, each contributing a small effect. Research has highlighted a positive genetic correlation between the predisposition toward autism and enhanced mental ability (31). Polimanti and Gelernter (40) argue that genomic data shows common alleles associated with increased AS risk, which underwent positive selection throughout human evolutionary history. Other evolutionary arguments for AS tie in with its polygenic genetic component, with at least 30 genes involved, suggesting autism is an unintended consequence of evolution (36).

Recent discussions surrounding double empathy and the double Theory of Mind paradigm have cast fresh illumination on the issue. Autistic individuals exhibit superior adaptability in empathizing with others within the spectrum compared to neurotypicals (41). They also demonstrate a noteworthy ability to comprehend animals, a trait potentially advantageous in their domestication (42). In addition, in hunter-gatherer communities heightened sensory sensitivity, often referred to as “oversensitivity,” could render autistic individuals vigilant hunters or sentinels (43). The prevalence of AS suggests the likely presence of some individuals in each of our ancestral hunter-gatherer groups. Some autistic individuals often demonstrate exceptional memory and spatial skills abilities and develop expertise in areas of particular interest. This may have conferred survival value in traditional societies, especially during food scarcity, where solitary foraging may have increased survival chances (44). For these reasons, the benefits of employing individuals with some forms of autism in various jobs have been increasingly appreciated.

Today, social communication deficits and repetitive behaviors seem to be more central to autism than previously thought, and these two aspects might be more fractionable than the 1979 triad concept suggests (45). In contrast with the classic view of a unitary autistic syndrome, the two components of the dyad are only weakly correlated with one another, both at the phenotypic and genotypic levels (46–48). Many sub-theories explain isolated phenomena in the autism spectrum, but only the Weak Central Coherence (WCC), Enhanced Perceptual Function (EPF), Bayesian Inference (BI), and Noisy Information Processing (NIP) are relevant to the compensation perspective presented here.

The WCC model refers to the ability to integrate individual pieces of information into a coherent whole (49). People with AS have a substantial bias toward the local processing of information which makes it harder to engage in global processing and see the “bigger picture” (50). This could explain why they often excel in tasks that require attention to detail but have difficulties in those that involve understanding of the overall context. WCC and an over-reliance on literal, detail-oriented processing may help to understand the difficulties with figurative language, such as figures of speech, jokes, and metaphors. Analysis of written and spoken language partially reveals a tendency to overinterpreting details and a reduced capacity for tolerating vagueness. This may reduce susceptibility to certain cognitive biases such as the framing effect (51) or optimism bias (52) while simultaneously impairing the effectiveness of particular problem-solving tactics when data is sparse. People with AS make consistent decisions (53) and have reduced susceptibility to conjunction fallacy or ultimatum game (54, 55).

The EPF model posits that compared to neurotypical individuals, people with autistic traits tend to rely more heavily on their perceptual processing rather than on higher-level cognitive processes like decision-making, planning, and problem-solving (56). This might also contribute to difficulties in communication, which often require the rapid and automatic integration of a wide range of cues and contexts that may be less tangible or less consistent than sensory information. Incorporating the relationship between low-level perceptual symptoms and higher-level cognitive abilities could also provide a more comprehensive view of the autism spectrum. This relationship suggests that while certain traits such as hypersensitivity can be challenging, they may also contribute to enhanced perceptual abilities. For instance, hypersensitivity to sensory input, often seen as a core symptom of autism, might also be linked to heightened attention to detail and superior memory skills (57). The co-occurrence of synesthesia and autism (58) further emphasizes the potential cognitive advantages that can arise from unique perceptual experiences. Synesthesia, a condition where sensory experiences are interconnected (e.g., seeing colors when hearing sounds), is more prevalent in individuals with autism. This overlap between autism and synesthesia suggests that the neural differences that underpin autism may also contribute to enhanced perceptual integration and creativity.

The BI approach to autism suggests that some of its core features might be related to differences in how the brain handles prediction and uncertainty. In a typical Bayesian brain model, prior beliefs (based on past experiences) are used to make predictions about the world, which are then compared to actual sensory input. The difference between the two is called the “prediction error.” The brain then updates its beliefs based on the prediction error. It is proposed that individuals with autism might have “weak priors,” meaning that their brains assign less weight to prior experiences and are thus more sensitive to incoming sensory information (59). This may explain why many individuals with autism are less able to filter out irrelevant sensory information (60), are more focused on details (since each piece of sensory information is considered highly informative) and have difficulties in social situations (which often involve complex and uncertain information). They require much more cognitive effort to update or change their beliefs than neurotypical individuals. This can lead to repetitive behaviors, routines, or interests, which all act as a compensation for sensory or processing overload that hinders flexibility.

The discrepancy regarding the BI model and the weighting of priors in AS is a point of ongoing debate. WCC suggests that AS give less weight to prior experiences, leading to a detail-focused cognitive style. Conversely, some research (61, 62), posits that AS might exhibit overreliance or inflexibility on prior expectations, which would imply a cognitive style influenced by strong, albeit less adaptable, priors. This apparent inconsistency can be reconciled by considering the context in which priors are applied and the heterogeneity of AS. The cognitive profile of AS is not uniform, and different environmental and developmental factors can lead to variations in how priors are weighted and used. One possible explanation for these divergent findings is the concept of context insensitivity, which posits that AS might struggle with adjusting their priors based on the context. This might manifest as weak priors when the situation requires integration of information for a “big picture” perspective, or as strong, inflexible priors in situations that require adaptation to new information or changing contexts.

According to NIP, individuals with autism have increased “neural noise” in their brains, which is essentially random activity or variability in neural networks. This might interfere with the brain’s ability to process sensory information, leading to less reliable and less consistent responses to stimuli. In practical terms, this could manifest in several ways, as: (1) increased sensory sensitivity and overload (2) difficulty ignoring irrelevant information (3) inconsistent performance on tasks (4) increased cognitive effort (62). Evidence shows that people with autistic-like traits systematically discount the weight of social information (63). Palmer et al. (64) proposed that autistic people lack the generic flexibility to adjust the weight of sensory inputs in a context-dependent fashion. A probable reason is that their weak priors have little predictive weight which is interpreted by the brain as supposedly overly stable, unchanging social environment. On this view, repetitive behaviors help reduce the person’s uncertainty about the environment at the expense of active, flexible exploration. In this line, they may be interpreted as a compensatory mechanism to cope with copious amount of sensory input.

Both WCC and EPF highlight the detail-oriented nature of autistic cognition. Computationally, this implies increased weights on local, immediate input or high-resolution perceptual processing. Evolutionarily, it might be advantageous in specific environments or tasks. The BI and WCC theories intersect when considering how weak priors might lead to a focus on local details. The EPF emphasis on enhanced perceptual abilities and the NIP focus on variability can intersect in explaining sensory sensitivities. Computationally, high-resolution perceptual processing combined with variability (noise) can lead to a system easily overwhelmed by sensory input. While noise can lead to overstimulation in specific social contexts, enhanced perception can aid in tasks requiring detailed attention.

Additional arguments in favor of our approach become apparent if we look at the temporal underpinning of these cognitive biases, which may shed additional light on this issue of social communication difficulties in general and the compensatory nature of repetitive behaviors in particular (65). The expansive body of studies concerning modifications in higher-order temporal processing, interval timing and time sensitivity within AS reveals their notable impact on intersubjective processes (66, 67). These perturbations can engender challenges in social synchronization (68) interpersonal communication (69), the recognition of causal relationships, the sense of agency (70), and the ascription of unity and continuity to experience (71). Higher-level temporal processing is responsible for understanding the abstract concept of time, placing oneself and events in the temporal continuum. Atypicalities in this domain may impede the ability to engage in planning and temporal orientation (72, 73). Interval timing and duration assessment is a skill essential for adjusting reaction times to specific stimuli and actions. Modifications within this temporal sphere can result in difficulties in foreseeing the sequence of steps within interpersonal communication. Time sensitivity encompasses simultaneity judgments (discriminating whether actions/events are simultaneous or sequentially connected) and temporal thresholds (assessing whether the subject perceives two equally enduring stimuli as possessing distinct temporal attributes). Simultaneity judgments are typically tied to the Temporal Binding Window, which integrates various stimuli into unified information. Difficulties within this realm may confuse the causal relationships of specific events and prospective timing. Experiencing stimuli as overlapping might evoke a sensation of being overwhelmed by the events.

Atypicalities in the aforementioned domains are often regarded as maladaptive traits. On the contrary, the compensation perspective highlights the potential advantages of such modifications. For example, half of the studies find a reduced susceptibility to negative biases in AS (74). Autism-specific atypicalities in higher-level temporal processing frequently led to the desynchronization of social processes (75, 76). In extreme cases, this may lead to isolation and challenges in adapting to the norms. It also results in reduced susceptibility to social influence, leading to the often reported “misunderstanding” of the rules of interpersonal interactions. This disadvantage, however, also makes decisions less burdened by biases stemming from social pressure and conformism toward a group (77, 78). The “weak priors” concept implies that decision-making processes are less encumbered by previous knowledge and more reliant on current information. Similarly, qualitative investigations of temporal experience in AS reveal a focus on the present moment “here and now” (79, 80). This suggests that prioritizing present data input over prior implicit knowledge (which is seen as “desynchronization”) can contribute to the reduction of implicit social biases in AS people (81).

Moreover, being anchored in the “here and now” can have the benefit of being able to analyze situations without being unduly influenced by past emotions. Contrary to stereotypes, autistic individuals often base their decisions on emotions, displaying “empathy-guided moral agency” similar to neurotypical persons (82). The divergence between these groups lies in the former’s lack of automatic linkage between the emotions accompanying past events, their relevance to the present situation, and the ability to project into the future. This temporal structure can impede the prediction of consequences. At the same time, the ability to disentangle turbulent past emotions from current decision process serves as a mechanism to circumvent cognitive distortions. For example, autistic individuals display greater resistance to the Sunk Cost Fallacy (83–85). Similarly, to WCC, the “expanded” Temporal Binding Window engenders slower integrative processing of perceptual information, leading to a sense of being overwhelmed by continuously overlapping events, creating a seamless “timescape.” The solution lies in creating a structured and predictable environment, coupled with routinized and repetitive behaviors and an analytical approach to understanding the situation.

Repetitiveness might be thus understood as a compensatory strategy aimed at counteracting the primary unpredictability by creating an “artificial time structure.” Well-structured behavioral routines make time more manageable for autistic individuals and mitigate the stress associated with novelty (79). Repetitive behaviors may be interpreted as adaptive strategies aimed at coping with predictability challenges (86). Vogel et al. (65) propose that an overly continuous passage of time contributes to the emergence of repetitive behaviors, which are postulated to represent a “forced division” of time through the use of stereotyped temporal intervals within activities. However, this trait might enable one to dwell in the here and now. Freed from excessive “forward thinking” they can allocate heightened attention and mental energy toward completing the ongoing task. The notably strong commitment to an activity is a characteristic feature of autistic individuals, granting them enhanced accuracy, speed, and precision in task performance, often surpassing that of neurotypical individuals.

While this characteristic, in its extreme form, can have drawbacks, it also might offer advantages. It is because innovativeness driving community development need not necessarily manifest as an unforeseen burst of genius-inspired enlightenment (87). Many historical inventions materialized through countless attempts to assemble various elements and implement incremental modifications until the most optimal configuration was achieved. From an evolutionary standpoint, the attributes that enabled individuals to sustain focus on repetitive activities for prolonged periods played a pivotal role in the development of human societies.

## Schizophrenia and empathizing: some subtypes of schizophrenia are unique cognitive strategy to handle social interactions at a price of overdetection of intentional behaviors

The DSM-5 (88) has abandoned categorizing schizophrenia into subtypes, instead opting for a dimensional assessment of primary symptoms. It outlines the diagnosis of schizophrenia based on the presence of delusions, hallucinations, disorganized thinking, abnormal motor behavior, and negative symptoms such as affective flattening. A diagnosis requires at least two of these symptoms, one of which must be delusions, hallucinations, or disorganized thinking, persisting for a minimum of 6 months.

Schizophrenia typically manifests in late adolescence or early adulthood, showing sex differences in onset with a male-to-female ratio of roughly 1.4:1 (89). Despite advancements in healthcare, individuals with schizophrenia have a life expectancy approximately 14.5 years less than the general populace (90). Nevertheless, the frequency of schizophrenia remains constant. There is also some limited GWAS (Genome-wide Association Studies) evidence pointing to the effect of negative selection of schizophrenia traits in humans in comparison to Neanderthals and Denisovans, which, in turn, suggests that schizotypal traits could be more beneficial in ancestral populations and are becoming increasingly maladaptive in the modern environment (91).

The Social Brain Theory postulates that the human brain has evolved specific mechanisms to perceive, interpret, and respond to complex social cues, facilitating group cohesion and survival. Disturbances in these mechanisms are thought to underpin the hallmark symptoms of schizophrenia. Understanding schizophrenia through the lens of the social brain provides new avenues for interventions. Targeting social cognitive deficits, such as Theory of Mind impairments, can be a fruitful approach for psychosocial therapies, aiming to enhance patients’ social functioning and thereby improving their quality of life (92). However, the Social Brain Theory lacks proper computational background, which is essential to construct a unified model of schizophrenia. From a computational standpoint, schizophrenia may arise from a neural imbalance where the brain overly relies on its internal predictions (strong priors) rather than new sensory input, resulting in perceptual anomalies, a concept supported by Bayesian Inference.

The human brain has experienced substantial growth, especially in regions like the neocortex, which is vital for higher cognitive functions including social cognition (93). Evolutionary biologists suggest that the increasing complexity of human social interactions drove the expansion and specialization of these brain regions, coining the term “social brain” (94). Social cognition encompasses various cognitive functions, such as recognizing social cues like facial expressions, eye movements, and body language, deducing others’ thoughts and feelings, evaluating others, and contemplating one’s relationship to them (95). Moreover, neuroimaging studies have consistently identified structural and functional abnormalities in the brain regions associated with social cognition in schizophrenia patients. These regions include the medial prefrontal cortex, anterior cingulate cortex, superior temporal sulcus, fusiform gyrus, and amygdala, all crucial for understanding others’ emotions, intentions, and beliefs (96, 97). One of the central components of social cognition is Theory of Mind (ToM), the ability to attribute mental states to oneself and others, predicting or explaining their behavior. Numerous studies have found that individuals with schizophrenia often display deficits in ToM tasks and metacognition in general, leading to difficulties in social interactions due to, e.g., overattribution of intentionality or mixing various levels of intentionality (98, 99). While there’s genetic susceptibility to schizophrenia, social factors play a significant role in its onset and progression. Stressful social experiences, such as childhood adversities or urban upbringing, can contribute to the development or exacerbation of schizophrenia, suggesting an intricate relationship between social brain dysfunctions and environmental factors (100). In full-blown schizophrenia, the symptoms often interfere with an individual ability to adapt to society. Moreover, some research suggests that certain schizotypal traits might be linked to heightened empathy or a more refined ability to “read” others. Positive schizotypy counterintuitively enhances social bonds, primarily through its beneficial impact on cognitive empathy (101). This increased sensitivity can be beneficial in navigating social hierarchies and understanding unspoken social cues, thus enhancing social cohesion. While pronounced schizophrenia can be disruptive, subclinical schizotypal traits might offer certain benefits in particularly chaotic social settings when viewed through an evolutionary lens. Nonetheless, it is essential to recognize that adaptivity can quickly turn to maladaptivity, depending on the context or intensity of the traits.

There are multiple facets of Schizophrenia Spectrum (SS) requiring distinct local subtheories. One of the important computational contributions to the evolutionary approach is the Disconnection Hypothesis suggesting that schizophrenia results from aberrant integration and coordination of neural circuits. The basis of this hypothesis is the dysfunction of synaptic plasticity leading to impaired connectivity in neural networks. This theory has been studied using computational neuroscience tools, including dynamic causal modeling and functional connectivity analysis (102, 103).

One interesting consequence of the Disconnection Hypothesis is the fact that people with schizophrenia often exhibit subtle disturbances in interpersonal relationships, communication, and affect. These disturbances may result from aberrant central integration and can manifest as unusual reactions, atypical affective responses, or a certain peculiarity in how they relate to others or perceive the world. Non-verbal communication, such as facial expressions, body language, or eye contact differ in people with schizophrenia. Psychiatrists become adept at picking up and interpreting these non-verbal and “bizarre” signals for diagnostic purposes, even if they cannot explicitly pinpoint why—this widely prevalent and cross-cultural phenomenon is known as the Praecox Feeling (104, 105).

Similarly, to AS, the computational approach to SS should be supplemented with the Bayesian Inference, which posits that schizophrenia is characterized by an imbalance between the brain’s predictions and the incoming sensory input. The brain in schizophrenia might give too much weight to its own internal predictions at the expense of new sensory information, leading to symptoms like hallucinations and delusions (106, 107). However, different initial assumptions might be set for various sensory inputs and their combination, and impairments in each sensory domain may vary in severity (108).

Additionally, Bayesian Inference suggests that brains operate as inference machines that constantly update a model of the world based on incoming sensory information (109, 110). However, in schizophrenia, this process might be globally impaired due to disconnection between certain brain regions leading to an over-reliance on prior beliefs or inadequate updating based on sensory evidence. Within the proposed framework, hallucinations could be understood as the brain relying too much on prior beliefs in the absence of confirmatory sensory input. The brain “hallucinates” what it expects to perceive based on its strong priors, even if no external stimulus supports that perception (111). Delusions can be conceptualized as the result of an over-weighting of prediction errors. When the brain misinterprets sensory input as being highly unexpected, it can form delusional beliefs to “explain” this unexpected input (112). Negative symptoms (affective flattening, alogia, anhedonia, avolition and asociality) could arise from a cognitive fatigue due to an overall brain overexcitation caused by positive symptoms. Strong priors may create an “alternate reality” (positive symptoms), which temporally exhausts cognitive reserves causing a compensatory underactive state (negative symptoms). If the brain does not sufficiently update its predictions based on sensory input due to cognitive fatigue, it could lead to a reduced engagement with the external world and a lack of motivation to act or feel (113). Bayesian approach could be also applied to understanding of the alterations in interoception and high-level integration between interoception and exteroception in schizophrenia (114) as well as atypical alternation in kinaesthesia and spatio-temporal experience (115).

Moreover, the Bayesian Inference provides a framework for understanding cognitive biases observed in SS—Jumping to Conclusions Bias, Bias Against Disconfirmatory Evidence, and Externalizing and Attribution Bias. Jumping to Conclusions Bias occurs where people make decisions based on minimal evidence. This can be seen as a result of over-reliance on prior beliefs and an under-weighting of new sensory evidence (116). In line with that reasoning, Bias Against Disconfirmatory Evidence occurs when individuals with schizophrenia presented with evidence that contradicts their beliefs tend to stick to their original belief. This can be understood as the brain giving too much weight to its priors and not enough to prediction errors (117). Lastly, the action of Externalizing and Attribution Bias could come into play due to people with schizophrenia attributing events to external forces rather than their own actions. Oversensitive theory of mind in the schizophrenia spectrum is often the cause of misattribution of intentional behavior even when taken to the extreme, like in the case of attributing intentions to inanimate objects, forces of nature or very abstract ontic instances, such as fate. This could be a result of the brain’s predictions (strong priors) being uncoordinated with neglected sensory input, leading to an external attribution for unexpected events (118).

In terms of neural connectivity, regions like the prefrontal cortex exhibit reduced connectivity to the thalamus in individuals with schizophrenia compared to controls, while primary sensory areas show increased connectivity with the thalamus (119). This consistent pattern aligns with findings suggesting that people with schizophrenia significantly devaluate future rewards more than healthy counterparts (120), with notable deficits in planning and impulse control (121). On a perceptual scale, there’s increased resilience against high-level visual illusions that leverage visual priors on ambiguous images. Moreover, there is an observable inability to reduce the sensory impact of one’s actions, potentially affecting the sense of agency (122).

The intersection of the Social Brain Theory, the Disconnection Hypothesis, and the Bayesian Inference in the context of schizophrenia offer a nuanced understanding of the condition rooted in both computational and evolutionary perspectives. An oversensitive theory of mind might be seen as an extreme manifestation of the evolved capacities to manage complex social interaction, leading individuals to perceive intentionality or agency where none exists, which can manifest as paranoid delusions or hallucinations (123). The Disconnection Hypothesis suggests that these symptoms arise due to faulty neural integration. This lack of effective communication between brain regions, especially those associated with social cognition and perception, could explain the difficulties schizophrenic patients face in distinguishing between self-generated thoughts and external voices or intentions. The Bayesian Inference posits that the brain operates as an inference machine, constantly updating its model of the world based on sensory information. In schizophrenia, this process could be impaired due to the aforementioned neural disconnections. Thus, patients may over-rely on prior beliefs (oversensitive theory of mind) and inadequately update these based on incoming sensory evidence, leading to misconstrued social perceptions.

## Conclusion: cognitive bias in autism spectrum and schizophrenia spectrum as a compensatory mechanism for specific ecological niches

The theoretical framework presented seeks to merge the computational approach, which emphasizes the brain’s information processing dynamics with the evolutionary perspective, which contextualizes these neural patterns within the broader canvas of human evolutionary history. In doing so, it casts autism and schizophrenia spectra not merely as anomalies but as manifestations of neurodiversity stemming from unique cognitive biases. Autistic individuals may overemphasize details and patterns, while schizophrenic individuals might over-attribute intentionality. While potentially maladaptive in contemporary environments, these biases might have held adaptive value in ancestral settings or under specific conditions. In the combined evolutionary and computational framework, understanding these biases is crucial. It allows researchers to decipher how ancestral pressures might have shaped these cognitive tendencies and how they manifest in modern neural circuits. Merging both perspectives paints a clearer picture of the genetic predispositions, neural circuits, and environmental factors at play. This integrative approach is critical to deciphering the diametrical model, emphasizing how two seemingly distinct disorders can share evolutionary and computational roots.

The proposed compensation perspective views autism as a unique intersection of cognitive biases and life history strategies, which are shaped by evolutionary pressures. The cognitive biases are based on WCC (focusing on details over the whole), EPF (focus on sensory input), NIP (increased default neural noise), BI bias (stronger weighting to sensory inputs than prior experiences), and reduced susceptibility to some above-mentioned cognitive biases. In this combined framework, all abovementioned cognitive mechanisms could be seen as an adaptive strategy in early human societies, particularly in environments where social change was slow, and social predictability was high. An enhanced ability to focus on details would be particularly advantageous in situations that required careful, meticulous attention, such as tool making or tracking game. In modern societies, these traits may contribute to some of the challenges of AS, such as difficulties with social communication or sensory hypersensitivity.

Both “weak priors” in AS and “strong priors” in SS create a subjective feeling of cognitive unexpectedness and can lead to processing difficulties. In AS the unexpectedness is caused by weak central coherence, weak priors, and overabundance of partially unstructured sensory input. Conversely, in SS it is caused by the lack of the integrity between strong priors and sensory input, aberrant central integration and overreactive cognitive biases. However, some forms of AS may be more functional in a very stable social environment (trustworthy people, highly structured daily routine, transparent social rules) where weak priors make it possible to extract hidden regularities and rules behind the natural world and, at least some, technical artifacts. Conversely, some forms of SS may be beneficial in a very unstable social environment, where social rules are constantly changing and people should not be trusted, e.g., due to harsh natural environmental conditions (social unrest, famine, plague, etc.). Cognitive biases thus emerge as pivotal concepts that bridge the gap between ancestral evolutionary pressures and contemporary cognitive manifestations. In sum, to truly grasp the complexities and nuances of the diametrical model of autism and schizophrenia, it is imperative to synergize the evolutionary and computational perspectives with the neurodiversity framework.

As a final note, the presented outlook invites a broader range of research questions, exploring not only the challenges but also the potential evolutionary advantages and societal contributions of individuals with these cognitive profiles. Investigating the optimal environmental conditions and support systems that allow individuals with AS and SS to thrive could lead to innovative treatment modalities and supportive technologies. Clinical interventions based on the evolutionary and computational perspectives outlined in the paper could focus on leveraging the specific cognitive biases and traits in AS and SS. Therapies for those who tend to over-rely on priors might focus on enhancing flexibility and adaptation to change. Conversely, for those who tend to underweight priors, therapies might focus on building connections between details and the broader context. Here are some examples of interventions that could be developed or refined with this approach.

### Cognitive remediation therapy

Cognitive remediation therapy (CRT) in AS could be tailored to help individuals better integrate details into a whole (addressing WCC) and improve social cognition by providing structured learning experiences that gradually increase in complexity. Similarly, CRT in SS could aim to stabilize the attribution of intentionality and reality monitoring, helping patients differentiate between internal thoughts and external stimuli.

### Sensory integration therapy

Sensory integration therapy (SIT) in AS could involve controlled exposure to various sensory stimuli to reduce hypersensitivity or hyposensitivity, using the evolutionary understanding of their sensory processing differences. Additionally, exploring the advantages of these perceptual phenomena can inform a strength-based approach in education and professional environments. Recognizing that individuals with autism might excel in tasks that require extraordinary attention to detail or pattern recognition could lead to more inclusive practices, such as job roles tailored to these strengths. However, in SS sensory therapies might focus on grounding techniques that help individuals reconcile strong priors with actual sensory input, thus reducing hallucinations or delusions.

### Strengths-based occupational therapy

Strengths-based occupational therapy (SBOT) in AS could focus on identifying environments or occupations where attention to detail and pattern recognition is advantageous, thus creating niches where individuals with AS can excel. Interventions in SS might center on harnessing creativity and divergent thinking, identifying careers or creative outlets where these traits are beneficial.

### Social skills training

Social skills training (SST) training in AS could include using predictable, rule-based social scenarios to practice communication, leveraging their tendency to thrive in structured environments. Analogously, in SS role-playing and simulation games might be used to help individuals understand and adapt to rapidly changing social dynamics.

### Environmental modifications

In case of AS it is necessary to create structured and predictable environments both at home and in educational or occupational settings to reduce anxiety and allow for greater focus on tasks. It may be useful to introduce moderate levels of variability in environments to help patients become more adaptable without becoming overwhelmed.

### Mindfulness and stress reduction

Both for AS and SS it may be beneficial to teach mindfulness-based stress reduction techniques that help manage anxiety and improve cognitive flexibility, tailored to each spectrum’s specific needs.

### Family and caregiver support

Providing education about the evolutionary and computational perspectives, helping caregivers to understand the value of cognitive diversity and to create supportive environments tailored to the individual’s strengths.

## Author contributions

MR: Conceptualization, Formal analysis, Investigation, Methodology, Resources, Validation, Writing – original draft, Writing – review & editing. MW: Conceptualization, Investigation, Methodology, Resources, Writing – original draft, Writing – review & editing. MM: Conceptualization, Data curation, Funding acquisition, Methodology, Project administration, Supervision, Validation, Writing – review & editing.
